# Fetal Megalencephaly with Cortical Dysplasia at 18 Gestational Weeks Related to Paternal UPD Mosaicism with *PTEN* Mutation

**DOI:** 10.3390/genes12030358

**Published:** 2021-03-02

**Authors:** Ritsuko Kimata Pooh, Megumi Machida, Issei Imoto, Eri Noel Arai, Hiroyasu Ohashi, Masayoshi Takeda, Osamu Shimokawa, Kaori Fukuta, Arihiro Shiozaki, Shigeru Saito, Hideaki Chiyo

**Affiliations:** 1 Fetal Diagnostic Center, CRIFM Clinical Research Institute of Fetal Medicine, Osaka 543-0001, Japan; machida.megumi@fetal-medicine-pooh.com (M.M.); chiyo.hideaki@fetal-medicine-pooh.jp (H.C.); 2 Clinical Laboratory, Ritz Medical Co., Ltd., Osaka 543-0001, Japan; ohashi.hiroyasu@ritz-med.com (H.O.); takeda.masayoshi@ritz-med.com (M.T.); shimokawa.osamu@ritz-med.com (O.S.); 3 Division of Molecular Genetics, Aichi Cancer Research Institute, Aichi 464-8681, Japan; issehgen@gmail.com; 4 Department of Obstetrics and Gynecology, University of Toyama, Toyama 930-0194, Japan; 20080004n@gmail.com (E.N.A.); kaorifu8348@gmail.com (K.F.); s33shio@med.u-toyama.ac.jp (A.S.); s30saito@med.u-toyama.ac.jp (S.S.)

**Keywords:** *PTEN*, uniparental disomy, SNP, microarray, BAF, mosaicism, megalencephaly, cortical dysplasia, fetus

## Abstract

The phosphatase and tensin homolog (*PTEN*) gene is a tumor-suppressor gene located on 10q22-23. Since the introduction of molecular genetics in prenatal diagnostics, various birth defects associated with gene mutations have been diagnosed. However, no reports on fetal cases related to *PTEN* mutation have been found, so far. We encountered a rare case of fetal *PTEN* mutation. Fetal macrocephaly was noted at 16 weeks. At 18 and 20 weeks, neurosonography revealed megalencephaly with an asymmetrical structure and multifocal polygyria. The head circumference (HC) was +6.2 SD at 18 weeks and +8.1 SD at 20 weeks. The parents opted for pregnancy termination, and the male fetus was delivered at 21 weeks, with HC +9.3 SD. Single-nucleotide polymorphism (SNP) array for amniotic cells showed paternal uniparental disomy (UPD) 10q mosaicism, and the mosaic ratio was calculated as 56% from B-allele frequency. Exome sequencing revealed the pathogenic *PTEN* mutation with mosaicism. The heterozygous *PTEN* mutation may not cause early manifestations from the fetal period, and an abnormal phenotype may appear after birth. This may be the reason why fetal defects associated with *PTEN* mutation are not detected. Since this case had homozygous and heterozygous mutations, survival was possible, exhibiting an incredibly huge head with cortical dysplasia from early pregnancy.

## 1. Introduction

The phosphatase and tensin homolog (*PTEN*) gene is a gene located on the long arm (10q22-23) of chromosome 10. Since *PTEN* is frequently found to have DNA mutations in various cancers, it has been called a tumor-suppressor gene. Germline mutation in *PTEN* causes congenital diseases, such as Cowden syndrome, Bannayan-Riley-Ruvalcaba syndrome, PTEN-related Proteus syndrome, and Proteus-like syndrome. These diseases are often associated with hamartoma and various cancers at high rates. Since the introduction of molecular genetics in prenatal diagnostics, various birth defects related to gene mutations have been diagnosed. However, no reports on early fetal cases associated with *PTEN* mutation have been found so far. We encountered a rare case, diagnosed using fetal neurosonography, single-nucleotide polymorphism (SNP) microarray, and exome sequencing. 

## 2. A Case and Genetic Test Method

**Figure 1:**
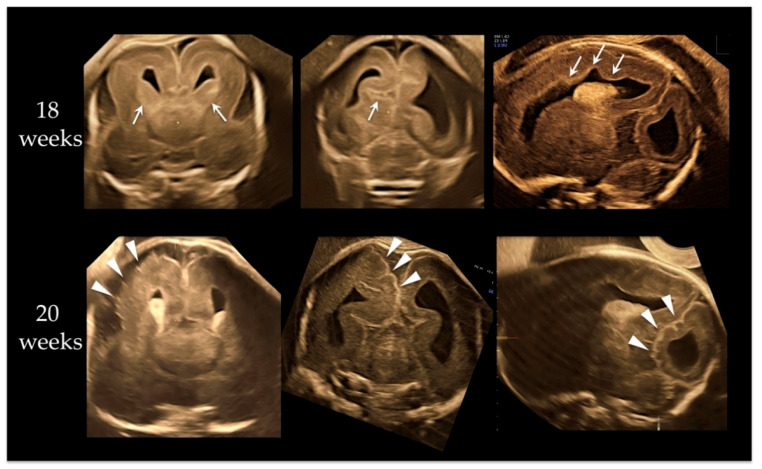
Neurosonographic findings at 18 and 20 weeks of gestation. Upper three images are at 18 weeks. From left, anterior coronal, posterior coronal, and parasagittal views. Megalencephaly with asymmetrical structure, broad ganglionic eminence (arrows on upper left image), abnormal sulcation (arrow on upper-middle image) on cerebral cortex, and irregular ventricular wall (arrows on upper right image) were demonstrated. Lower three images are at 20 weeks. From left, anterior coronal, posterior coronal, and parasagittal views. A remarkable cortical malformation with multifocal polygyria (arrowheads) was visualized; polygyria were not seen at 18 weeks. Compared to upper images, taken two weeks before, focal cortical dysplasia due to rapid neuronal migration disorder may have occurred randomly in various cerebral cortex regions.

**Figure 2:**
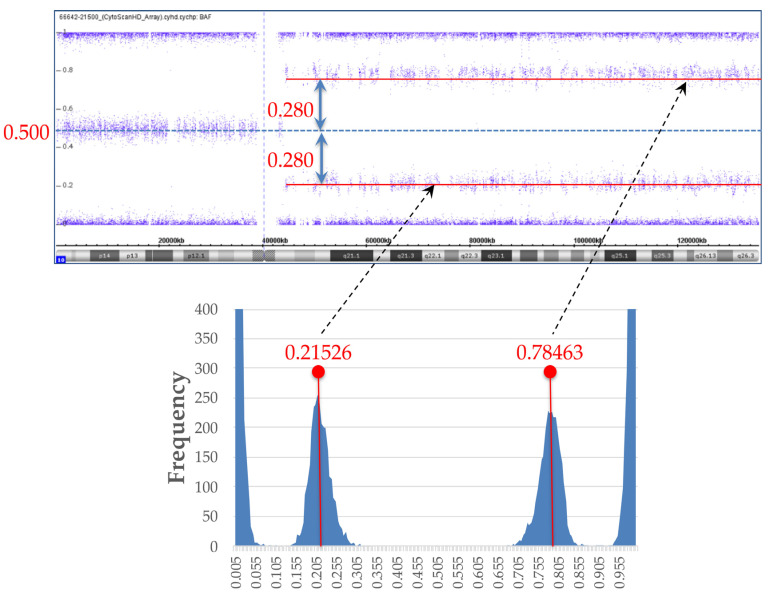
B-allele frequency (BAF) data and histogram from BAF data. For investigation of mosaic ratio, histogram was created from BAF data of single-nucleotide polymorphism (SNP) microarray (lower graph). Medians of two peaks in histogram were calculated, with BAF values of 0.21526, and 0.78463, respectively. Difference between peak median BAF value and BAF value of heterozygous SNPs of 0.5 was ±0.280.

A 37-year-old primipara was referred at 18 weeks due to macrocephaly noted at 16 gestational weeks. The pregnancy course was not eventful, and no particular family history existed. Fetal ultrasound demonstrated fetal macrocephaly with a 58.4 mm (+6.1 SD) biparietal diameter (BPD) and 213.6 mm (+6.2 SD) head circumference (HC). Neurosonography revealed megalencephaly with asymmetrical structure, broad ganglionic eminence, abnormal sulcation on the cerebral cortex, and irregular ventricular wall, as shown in the upper images of Figure 1. We conducted amniocentesis for genetic testing. We performed neurosonography again at 20 weeks of gestation. BPD and HC became 68.0 mm (+6.9 SD) and 254.9 mm (+8.1 SD), respectively. A remarkable cortical malformation with multifocal polygyria appeared, as shown in lower images of Figure 2. The polygyria were not depicted at 18 weeks. Compared to the upper images, taken two weeks earlier, multifocal cortical dysplasia due to rapid neuronal migration disorder may have occurred randomly in various cerebral cortex regions. The parents opted for pregnancy termination due to the high probability of an adverse postnatal neurological prognosis. The male fetus weighed 724 g (+3.9 SD) with a 280 mm HC (+9.3 SD) at 21 weeks and five days. No external malformations were observed other than the huge head. Postmortem autopsy was not performed because the parents did not permit it.

### 2.1. DNA Extraction and Routine Protocol of Genetic Tests

Amniotic fluid cells obtained at 18 gestational weeks were immediately divided into 2 groups, one for long-term culture and the other for DNA extraction. Genomic DNA (gDNA) was directly extracted using DNeasy^®^ Blood and Tissue Kit (Qiagen, Hilden, Germany) from 20 mL of amniotic fluid and 10 mL of parental blood samples. The gDNA was divided into a portion for QF-PCR and a portion for subsequent tests. QF-PCR was performed first to detect 3 significant trisomies (13, 18, and 21) and sex chromosome aneuploidy using an AnueFast^TM^ QF-PCR Kit (Genomed Ltd., Kent, UK), following the manufacturer’s protocol, and SNP microarray. The remaining gDNA was preserved for further genetic tests. The sample that remained after extracting the DNA was long-term cultured and subjected to G-banding for karyotyping.

### 2.2. SNP Microarray

The trio-based SNP microarray was performed with CytoScan^TM^ HD Array (Affymetrix, Santa Clara, CA, USA) following the manufacturer’s instructions. Subsequent data analysis was performed with Chromosome Analysis Suite (ChAS; Affymetrix, Santa Clara, CA, USA), and the output data were compared with the Database of Genomic Variants (DGV; http://dgv.tcag.ca/dgv/app/home (accessed on 1 February 2019)) and DECIPHER (https://decipher.sanger.ac.uk (accessed on 1 February 2019)). 

In relation to the mosaic ratio, we created a histogram from the B-allele frequency (BAF) data of the SNP microarray and calculated the cell proportion with mosaicism from the difference between B deviation and an expected BAF value of 0.5 for heterozygous SNPs. 

### 2.3. Exome Sequencing

Trio-based targeted next-generation sequencing (NGS) with the TruSight One Sequencing Panel (4813 genes) was conducted following the manufacturer’s instructions (Illumina Inc., San Diego, CA, USA). Prepared libraries were run on the Illumina MiSeq platform (Illumina Inc., San Diego, CA, USA) for 151 bp paired-end reads. After variant calling with HaplotypeCaller, putative diagnostic variants were extracted through an automated variant filtering pipeline. Then functional prediction of remained variants was performed by multiple algorithms with dbNSFP v4.0–4.1. Subsequently, flagged variants were manually reviewed and validated based on the American College of Medical Genetics (ACMG) guideline for interpreting sequencing variants. The putative variant was confirmed by Sanger sequencing. Finally, the flagged variant was comprehensively evaluated by comparing the patient’s clinical manifestations and family history with previously reported phenotypes and clinical records regarding putative variants.

## 3. Result of Genetic Tests

We obtained normal QF-PCR and G-banding results. The karyotype was 46, XY normal male. SNP microarray resulted in partial UPD mosaicism. However, it was difficult to explain the fetal brain abnormalities due to UPD mosaicism. Then, we proceeded with exome sequencing.

### 3.1. SNP Microarray and BAF Analysis for Mosaic Ratio

SNP array resulted in arr[hg19] 10q11.21q26.3(43,861,923–135,426,536) hmz(mosaic), indicating paternal UPD 10q mosaicism. We created a histogram from the BAF data of SNP microarray to investigate the mosaic ratio, as shown in Figure 2. We calculated the medians of two peaks in the histogram; their BAF values were 0.21526, and 0.78463, respectively. The difference between the peak median BAF value and the BAF value of heterozygous SNPs of 0.5 was ±0.280 (Figure 2). 

**Figure 3:**
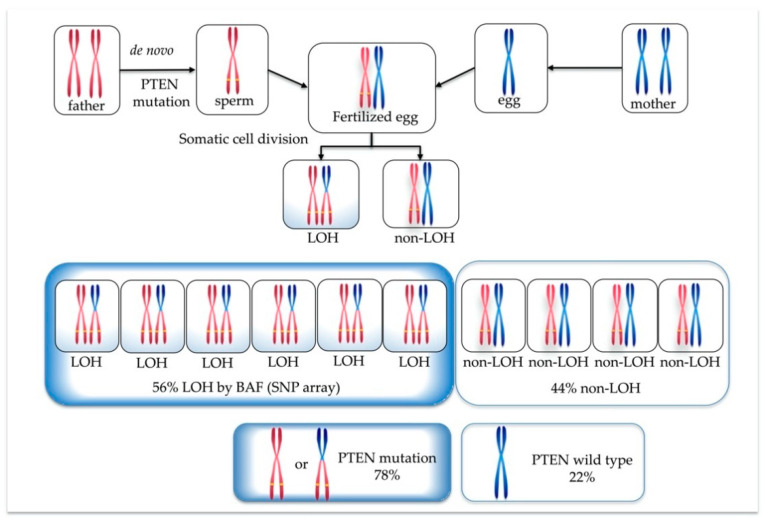
Origin of each chromosome, ratio of loss of heterozygosity (LOH) (homozygous *PTEN* mutation)/non-LOH (heterozygous *PTEN* mutation) cells, and percentage of *PTEN* mutated/wild-type alleles.

From the BAF analysis result, the value obtained by doubling 0.28 became the mosaic cell ratio. Thus, the mosaic cell proportion of loss of heterozygosity (LOH) containing homozygous *PTEN* mutation was 56%. The remaining 44% of cells were estimated to be non-LOH with heterozygous *PTEN* mutation. Figure 3 illustrates each chromosome’s origin, the ratio of LOH (homozygous mutation)/non-LOH (heterozygous mutation) cells, and the rate of *PTEN* mutated/wild-type alleles. A *PTEN* mutated allele ratio of 78% was calculated by the following formula: (56 × 2 + 44)/200 = 78.

### 3.2. Exome Sequencing

**Figure 4:**
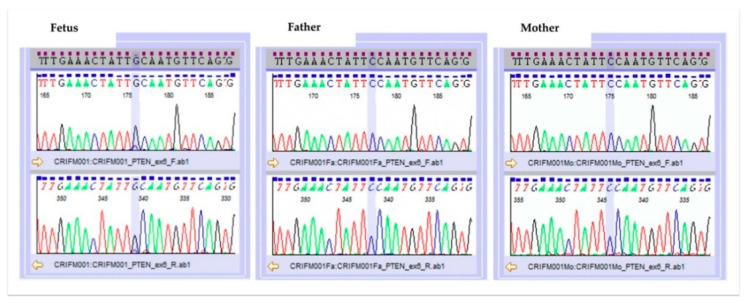
Result of Sanger sequencing.

A pathogenic missense mutation was found in exon6 of the *PTEN* gene (NM_000314.6), with mosaic rate of approximately 70%. This result was confirmed by Sanger sequencing (Figure 4). This mutation was not present in either the father or the mother. The detected gene mutation was not registered in the polymorphism database (dbSNP138, 1000Genome, iJGVD), while in the mutation database (HGMD, ClinVar), Pro204Ser is suspected to be a pathogenic mutation at the same codon. Developmental delays, autism, and macrocephaly have been reported for point mutations in adjacent amino acids [1]. In functional prediction, the point mutation causes highly conserved amino acid substitutions or splice abnormalities that become disease-causing or deleterious. In the heterozygous case, it is a germline *PTEN* mutation that shows a broad phenotypic spectrum, but in this case, homozygous dysfunction may have led to the prominent phenotype. Overall, the NGS result was pathogenic according to the American College of Medical Genetics (ACMG) guideline [2] for interpreting sequence variants.

Table 1 shows the *PTEN* mutation and wild-type allele ratio calculated from the BAF data of SNP microarray and next-generation sequencing (NGS) data. These were calculated from a completely different approach, but roughly similar ratios were found.

## 4. Discussion

The *PTEN* gene is a tumor-suppressor gene on the long arm of chromosome 10. PTEN inhibits PI3K-dependent signaling and regulates cell growth and survival. PTEN also inhibits Akt by suppressing the activity of PI3K-mediated signal transduction [3,4,5,6,7]. That is, PTEN negatively regulates invasion, migration, and cell survival. Germline mutations in the *PTEN* gene are responsible for several syndromes that result in multiple hamartoma lesions derived from the three germ layers. A disorder called PTEN hamartoma tumor syndrome (PHTS) [8,9,10,11,12,13] has been proposed.

The *PTEN* gene plays an essential role in neurodevelopment. In the first trimester of normal pregnancy, PTEN immunoreactivity in the developing human neocortex expresses strongly, then gradually decreases during the fetal period. On the contrary, Akt level increases during pregnancy [14]. The most common clinical manifestation with mutated *PTEN* is macrocephaly [15]. Volume increase due to overgrowth of intracranial structure causes the head circumference to increase. It has been described that *PTEN* loss in neuroglial progenitor cells of the central nervous system at mid-gestation causes increased progenitor cell numbers due to increased proliferation and decreased apoptosis [16]. An experimental report with knockout mouse models showed that cortical weight significantly increased in the homozygous mutated *PTEN* model [17]. In another report, cortex thickness did not change among control, heterozygous *PTEN* mutation, and homozygous *PTEN* mutation groups [18]. White matter abnormalities [9,19], and vascular abnormalities [20], are common. 

Although it had been described that cortical dysplasia with *PTEN* mutation was a rare finding, some cases were recently reported [21,22,23]. Hemimegaloencephaly with pachygyria [22] and focal cortical dysplasia with abnormal sulcation [23] were observed in patients with *PTEN* mutations. The incidence of cortical dysplasia in humans suggests that the *PTEN* gene is involved in neuronal migration [24]. Prominent multifocal cortical dysplasia was present in our case at 18–20 weeks of gestation. In our case, the apparent megalencephaly already existed at 18 weeks and was overgrowing. Head circumference increased from +6.2 SD at 18 weeks to +8.1 SD at 20 weeks and +9.2 SD at 21 weeks of gestation. At 18 weeks, the brain structure was asymmetrical, and there were findings such as residual basal ganglia and irregular ventricular surface (Figure 1), predicting the future cortical maldevelopment. Twenty weeks is the fetal age when cerebral gyration is not yet formed in normal cases, but multifocal polygyria were visualized randomly in various cerebral cortex regions. Those findings indicate that *PTEN* mutation may be deeply involved in neuronal migration disorder as well as cell proliferation. 

**Figure 5:**
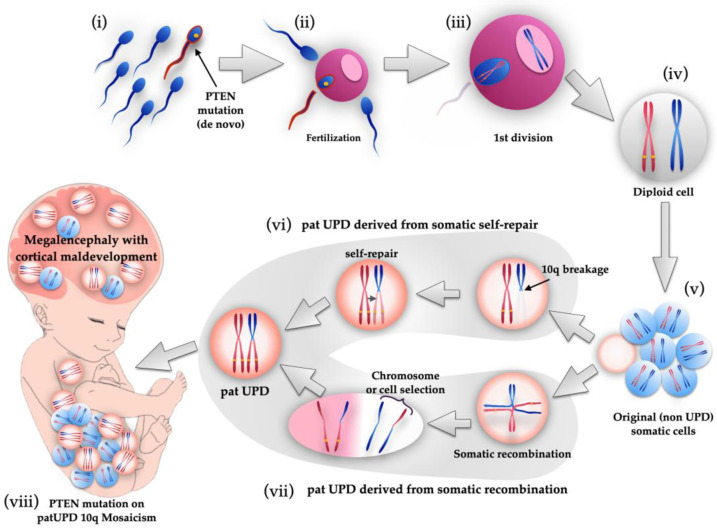
Schematic illustration of genetic events of fetus with *PTEN* mutation on paternal UPD 10q mosaicism. First, de novo *PTEN* mutation occurred in a sperm (**i**), and it fertilized an egg (**ii**). After first division (**iii**), diploid cell was created with *PTEN* mutated paternal and wild-type maternal chromosomes (**iv**). After that, somatic cells proliferated by mitosis, but UPD mosaicism cells were not yet produced at this stage (**v**). During mitosis, two possible genetic events cause the appearance of paternal UPD cells. One theory is 10q breakage and self-repair (**vi**). Another theory is somatic recombination and subsequent chromosome selection or cell selection (**vii**). Therefore, a fetus with paternal UPD 10q mosaicism with *PTEN* mutation was created, manifesting megalencephaly and cortical maldevelopment (**viii**).

We calculated the mosaic ratio from BAF data obtained from the SNP microarray result, as in oncogene diagnosis [25,26,27]. As shown in Table 1, both BAF and NGS data revealed that 70–78% of mutated and 22–30% of wild-type alleles were present. Based on the data, we speculated on how the *PTEN* mutation would occur in this case. Since it was confirmed that the wild-type came from the father, from the trio-based NGS result, it is difficult to assume that wild-type zygotes were fertilized, LOH occurred during mitosis, and then some cells selectively developed *PTEN* mutation. If the father-derived zygote already had a *PTEN* mutation before fertilization, it could be explained that the fetus formed with *PTEN* mutation at the ratio shown in Figure 3 and Table 1. Therefore, we concluded that the *PTEN* mutation, in this case, occurred in the sperm, and a sperm with the mutation happened to fertilize successfully, as shown in Figure 5.

Some reports suggest that the phenotype due to *PTEN* abnormality may have already occurred in the fetal period [28], but this is probably the first report that reveals the phenotype due to mutated *PTEN* in the first half of pregnancy. There has been no fetal case report because the heterozygous *PTEN* mutation does not exhibit a prominent phenotype during the fetal period. Our case was accompanied by extremely abnormal megalencephaly and cerebral cortex dysplasia in the first half of pregnancy, probably because homozygous cells with *PTEN* mutation accounted for 56% due to the existence of paternal 10q UPD mosaicism. In other words, a fetus with completely heterozygous cells theoretically has 50% of mutated *PTEN* allele, but in this case, it was as high as 70–78%. This indicates that heterozygous *PTEN* mutation may not cause the early manifestation from the fetal period, and abnormal phenotype may appear after birth. On the contrary, if we assume that all cells are homozygous, early fetal demise may occur in early pregnancy. It was reported that mice with homozygous *PTEN* loss died before embryonic day 7.5, since the tissue could not appropriately differentiate into three germ layers of endoderm, mesoderm, and ectoderm, while mice with the heterozygous mutated *PTEN* allele were viable and showed neuronal proliferation during brain development [24,29].

Since this case was a mosaic mutant mixed with both homozygous and heterozygous alleles, survival was possible, exhibiting an incredibly huge head with cortical dysplasia from early pregnancy.

## 5. Conclusions

PTEN plays a vital role in regulating embryonic/fetal development from early pregnancy, thereby, controlling neuronal cell proliferation and cell fate. This case report may help determine the relationship between the timing of phenotype expression and the mutated *PTEN* ratio.

## Figures and Tables

**Table 1:** Ratio of *PTEN* mutation and wild-type alleles from SNP array and NGS data.

Data Origin	*PTEN* Mutation	*PTEN* Wild-Type
BAF data (SNP array)	78%	22%
NGS data	70%	30%

## Data Availability

The datasets presented in this study are available from the corresponding author. The data are not publicly available due to the individual information.

## References

[1] Varga E.A., Pastore M., Prior T., Herman G.E., McBride K.L. (2009). The prevalence of PTEN mutations in a clinical pediatric cohort with autism spectrum disorders, developmental delay, and macrocephaly. Genet. Med..

[2] Richards S., Aziz N., Bale S., Bick D., Das S., Gastier-Foster J. (2015). Standards and Guidelines for the Interpretation of Sequence Variants: A Joint Consensus Recommendation of the American College of Medical Genetics and Genomics and the Association for Molecular Pathology. Genet. Med..

[3] Mirzaa G.M., Rivière J.B., Dobyns W.B. (2013). Megalencephaly syndromes and activating mutations in the PI3K-AKT pathway: MPPH and MCAP. Am. J. Med. Genet. Part C Semin. Med. Genet..

[4] Lugo J.N., Smith G.D., Arbuckle E.P., White J., Holley A.J., Floruta C.M., Ahmed N., Gomez M.C., Okonkwo O. (2014). Deletion of PTEN produces autism-like behavioral deficits and alterations in synaptic proteins. Front. Mol. Neurosci..

[5] Rivière J.B., Mirzaa G.M., O’Roak B.J., Beddaoui M., Alcantara D., Conway R.L., St-Onge J., Schwartzentruber J.A., Gripp K.W., Nikkel S.M. (2012). De novo germline and postzygotic mutations in AKT3, PIK3R2 and PIK3CA cause a spectrum of related megalencephaly syndromes. Nat. Genet..

[6] Shen W.H., Balajee A.S., Wang J., Wu H., Eng C., Pandolfi P.P., Yin Y. (2007). Essential Role for Nuclear PTEN in Maintaining Chromosomal Integrity. Cell.

[7] Pal A., Barber T.M., Van de Bunt M., Rudge S.A., Zhang Q., Lachlan K.L., Cooper N.S., Linden H., Levy J.C., Wakelam M.J.O. (2012). PTEN Mutations as a Cause of Constitutive Insulin Sensitivity and Obesity. N. Engl. J. Med..

[8] Shiohama T., Levman J., Vasung L., Takahashi E. (2020). Brain morphological analysis in PTEN hamartoma tumor syndrome. Am. J. Med. Genet. Part A.

[9] Balci T.B., Davila J., Lewis D., Boafo A., Sell E., Richer J., Nikkel S.M., Armour C.M., Tomiak E., Lines M.A. (2018). Broad spectrum of neuropsychiatric phenotypes associated with white matter disease in PTEN hamartoma tumor syndrome. Am. J. Med. Genet. Part B Neuropsychiatr. Genet..

[10] Mester J.L. (2016). PTEN hamartoma tumor syndrome. Intestinal Polyposis Syndromes: Diagnosis and Management.

[11] Mester J.L., Tilot A.K., Rybicki L.A., Frazier T.W., Eng C. (2011). Analysis of prevalence and degree of macrocephaly in patients with germline PTEN mutations and of brain weight in Pten knock-in murine model. Eur. J. Hum. Genet..

[12] Plamper M., Gohlke B., Schreiner F., Woelfle J. (2019). Phenotype-driven diagnostic of PTEN hamartoma tumor syndrome: Macrocephaly, but neither height nor weight development, is the important trait in children. Cancers.

[13] Michaela P., Mark B., Bettina G., Felix S., Sandra S., Vera S., Joachim W. (2020). Cerebral MRI and Clinical Findings in Children with PTEN Hamartoma Tumor Syndrome: Can Cerebral MRI Scan Help to Establish an Earlier Diagnosis of PHTS in Children?. Cells.

[14] Tanriover G., Demir N., Pestereli E., Demir R., Kayisli U.A. (2005). PTEN-mediated Akt activation in human neocortex during prenatal development. Histochem. Cell Biol..

[15] Hansen-Kiss E., Beinkampen S., Adler B., Frazier T., Prior T., Erdman S., Eng C., Herman G. (2017). A retrospective chart review of the features of PTEN hamartoma tumour syndrome in children. J. Med. Genet..

[16] Fraser M.M., Zhu X., Kwon C.H., Uhlmann E.J., Gutmann D.H., Baker S.J. (2004). Pten loss causes hypertrophy and increased proliferation of astrocytes in vivo. Cancer Res..

[17] Tilot A.K., Gaugler M.K., Yu Q., Romigh T., Yu W., Miller R.H., Frazier T.W., Eng C. (2014). Germline disruption of Pten localization causes enhanced sex-dependent social motivation and increased glial production. Hum. Mol. Genet..

[18] Frazier T.W., Embacher R., Tilot A.K., Koenig K., Mester J., Eng C. (2015). Molecular and Phenotypic Abnormalities in Individuals with Germline Heterozygous PTEN Mutations and Autism. Mol. Psychiatry.

[19] Vanderver A., Tonduti D., Kahn I., Schmidt J., Medne L., Vento J., Chapman K.A., Lanpher B., Pearl P., Gropman A. (2014). Characteristic brain magnetic resonance imaging pattern in patients with macrocephaly and PTEN mutations. Am. J. Med. Genet. Part A.

[20] Tan W.H., Baris H.N., Burrows P.E., Robson C.D., Alomari A.I., Mulliken J.B., Fishman S.J., Irons M.B. (2007). The spectrum of vascular anomalies in patients with PTEN mutations: Implications for diagnosis and management. J. Med. Genet..

[21] O’Rourke D., Twomey E., Lynch S.-A., King M. (2012). Cortical dysplasia associated with the PTEN mutation in Bannayan Riley Ruvalcaba syndrome. Clin. Dysmorphol..

[22] Jansen L.A., Mirzaa G.M., Ishak G.E., O’Roak B.J., Hiatt J.B., Roden W.H., Gunter S.A., Christian S.L., Collins S., Adams C. (2015). PI3K/AKT pathway mutations cause a spectrum of brain malformations from megalencephaly to focal cortical dysplasia. Brain.

[23] Child N.D., Cascino G.D. (2013). Mystery case: Cowden syndrome presenting with partial epilepsy related to focal cortical dysplasia. Neurology.

[24] Skelton P.D., Stan R.V., Luikart B.W. (2019). The Role of PTEN in Neurodevelopment. Mol. Neuropsychiatry.

[25] Farrar J.E., Vlachos A., Lipton J.M., Auerbach A.D. (2013). Arrays and the Cumulative Distribution Function. Mol. Genet. Metab..

[26] Rodríguez-Santiago B., Malats N., Rothman N., Armengol L., Garcia-Closas M., Kogevinas M., Villa O., Hutchinson A., Earl J., Marenne G. (2010). Mosaic uniparental disomies and aneuploidies as large structural variants of the human genome. Am. J. Hum. Genet..

[27] Attiyeh E.F., Diskin S.J., Attiyeh M.A., Mossé Y.P., Hou C., Jackson E.M., Kim C., Glessner J., Hakonarson H., Biegel J.A. (2009). Genomic copy number determination in cancer cells from single nucleotide polymorphism microarrays based on quantitative genotyping corrected for aneuploidy. Genome Res..

[28] Tekin M., Hismi B., Fitoz S., Yalcınkaya F., Ekim M., Kansu A., Ertem M., Deda G., Tutar E., Arsan S. (2006). A Germline PTEN Mutation With Manifestations of Prenatal Onset and Verrucous Epidermal Nevus. Am. J. Med. Genet. Part A.

[29] Cristofano A.D., Pesce B., Cordon-Cardo C., Pandolfi P.P. (1998). Pten is essential for embryonic development and tumour suppression. Nat. Genet..

